# Sex-Specific DNA Methylation in the Liver of *Ancherythroculter nigrocauda*, a Fish with Female-Biased Size Dimorphism

**DOI:** 10.3390/ani16203300

**Published:** 2026-10-20

**Authors:** Jian Chen, Pei Li, Lifei Luo, Hongxi Guo, Yanhong Sun

**Affiliations:** Fisheries Research Institute, Wuhan Academy of Agricultural Sciences, Wuhan 430207, China; chenjian@wuhanagri.com (J.C.); lipei@wuhanagri.com (P.L.); luolifei@wuhanagri.com (L.L.); guohongxi@wuhanagri.com (H.G.)

**Keywords:** sexual size dimorphism, DNA methylation, liver, gene expression, correlation

## Abstract

Sexual size dimorphism (SSD) is widespread in teleost fishes, yet the epigenetic mechanisms underlying sex-specific growth remain poorly understood, particularly in non-model species. Here, we performed genome-wide DNA methylation profiling of liver tissue in *Ancherythroculter nigrocauda*, a species exhibiting female-biased SSD, using the MethylRAD approach. MethylRAD sequencing of five females and five males generated approximately 1.0–1.5 million unique methylated loci per sample. Comparative analysis identified 82,872 differentially methylated sites (DMSs), which clearly separated sexes and were significantly enriched in promoters and CpG islands. Gene ontology analyses revealed that sex-biased methylation preferentially affected pathways related to intracellular signaling, cell adhesion, cytoskeletal organization, and protein modification, processes closely associated with hepatic growth and metabolic regulation. Chromosomal binning uncovered large contiguous male-biased methylation domains on Superscaffold15 and Superscaffold20, consistent with recombination-suppressed regions and suggesting candidate sex-linked loci, with Superscaffold20 supported by prior sex-specific coverage differences. Integration with liver transcriptome data revealed a threshold-dependent regulatory architecture: genes harboring dense methylation clustering (>20 DMS) were approximately six-fold more likely to exhibit sex-biased expression, and directional concordance between methylation and transcription further supported functional coupling. Together, these findings demonstrate that sex-biased DNA methylation in the liver is widespread yet concentrated within regulatory hotspots and discrete chromosomal domains, highlighting epigenetic regulation as a key contributor to hepatic sexual dimorphism and providing new insights into the molecular basis of SSD in teleost fishes.

## Simple Summary

Sex differences in body size are common in fish, but the biological mechanisms underlying these differences remain poorly understood. *Ancherythroculter nigrocauda* is a freshwater fish, and females grow substantially larger than males, with mature females reaching two- to four-times the body weight of males. Understanding the mechanisms responsible for this difference is important for improving our knowledge of fish growth and for aquaculture production. In this study, we examined DNA modification patterns in the liver of male and female fish to investigate whether these patterns are associated with sex-related differences in growth and liver function. We identified 82,872 DNA modification sites that differed between males and females. These differences were concentrated in regions that can regulate gene activity and were associated with biological processes involved in cell communication, metabolism, protein processing, tissue organization, and growth. Genes with a high number of DNA modification differences were also more likely to show sex-related differences in gene activity, suggesting that extensive DNA modification may contribute to differences between the sexes. We further identified two genomic regions with sex-related methylation patterns that may be associated with sex determination. Overall, our findings suggest that DNA modification is an important regulatory mechanism underlying sex-related differences in liver function and growth in this species, providing useful information for understanding sexual differences in fish and their potential application in aquaculture.

## 1. Introduction

Sexual dimorphism (SD) refers to the differences between males and females in forms of significant anatomical, physiological, and behavioral traits [1]. These differences might offer genetic fitness, which promotes survivability when the species is subjected to evolutionary selection [2]. In teleost fish, SD showed the difference in various aspects, including body size, body color, habitat, prey, or behavior. Sexual size dimorphism (SSD) is one of the most common and striking SDs in various fishes, and it can be observed as male-biased or female-biased [3,4]. For fishes with male-biased SSD, larger males might be favored by natural selection and/or sexual selection in preying and reproduction [1,5]. Under a constraint-free model, larger males are more likely to access more mates and, therefore, have a higher level of spawning than smaller males [1]. For female-biased fish with SSD, females grow faster and mature with a larger size than males. One hypothesis for females’ larger size is the survival of fry is mainly decided by the number rather than the quality of eggs [6]. Though SD is widespread in teleost fish, knowledge about the mechanism and origin of SD is still in its infancy [7].

*Ancherythroculter nigrocauda* belongs to the cyprinid subfamily Cultrinae, an important component of the freshwater ichthyofauna of China [8]. This species exhibits pronounced female-biased SSD, with females growing faster and reaching approximately 2–4-times the body weight of males [9]. Larger mature size is of great importance and economic value in aquaculture. Many studies have been conducted in female-biased aquaculture species about the mechanism of SSD [10,11]. In half-tongue sole (*Cynoglossus semilaevis*), each female has a body size of up to about four-times that of a male. RNA-Seq of four tissues between males and females was conducted, and lots of sex-related differentially expressed genes (DEGs) were found [4]. RNA-Seq was also used between male and female olive flounder (*Paralichthys olivaceus*) for the identification of potential SSD-related genes [11]. A previous study in our lab found that genes associated with cytochrome P450, PPAR signaling and steroid hormone biosynthesis were enriched in differentially expressed genes between female and male *Ancherythroculter nigrocauda* [9]. In addition, transcriptome profile studies were also used in male-biased fish with SSD, which proved it as a vital method in studying the mechanism of SSD [12,13].

In addition to genetics, epigenetics has also been found to be responsible for the establishment of SSD [14]. DNA methylation is a well-known epigenetic modification, and CpG methylation in the most common and studied form of DNA methylation [15]. There is evidence that CpG methylation has a major role in creating and establishing sex-specific differences [16,17]. In olive flounders, different DNA methyltransferase gene family members showed distinct expression pattern between ovary and testis, which indicated the different pattern of DNA methylation in SD [18]. In tilapia, whole-genome bisulfite sequencing (WGBS) was used to study the relationship between methylation and SD, and the MAPK/ERK pathway was found to be upregulated in male tilapia [14]. There are also studies focused on specific genes’ epigenetic modification on sex-related genes in fishes with SSD [19,20].

In this study, we applied methylation-sensitive sequencing, MethylRAD, on *Ancherythroculter nigrocauda* liver tissue to investigate the whole-genome DNA methylation landscape. The liver integrates various hormone signals triggered by alterations in metabolism and acts as a critical regulator of metabolism homeostasis [21]. And it sustains SSD by influencing the hypothalamic–pituitary–adrenal (HPA) axis [21]. Here, we report the first methylome of liver tissue, including sexually dimorphic differentially methylated regions (DMRs). This study will provide more knowledge for the regulatory role of epigenetics in SSD.

## 2. Materials and Methods

### 2.1. Ethics Statement

This study was conducted in accordance with applicable institutional and national guidelines for animal research and the ARRIVE guidelines 2.0. The fish samples used in this study were originally collected for our previous research published in 2021 [9]. The original fish collection and experimental procedures were approved by the Animal Care and Use Committee at the Wuhan Academy of Agricultural Sciences (protocol AA_2019_0012, approved on 17 May 2019). No additional fish were collected or sacrificed specifically for the present study. The overall research design and subsequent analyses of the current study were approved by the same committee (protocol AA_2024_0049, approved on 20 April 2024).

### 2.2. MethylRAD Library Preparation and Sequencing

The liver tissues used in the present study were obtained from the same individual fish analyzed in our previous transcriptomic study [9]. In brief, liver tissues of 5 females and 5 males at sexual maturity (24 months) were collected in our breeding fish farm (Wuhan, China). The mean body weight for female samples was 575.8 ± 56.8 g and male samples 138.8 ± 7.0 g. This sample size was selected to provide biological replication while maintaining sufficient sequencing depth for genome-wide methylation profiling. In addition, the same individuals had been characterized in our previous transcriptomic study [9], enabling direct integration of methylation and gene expression data without introducing inter-individual variation between the two datasets. Genomic DNA was extracted from liver tissues using an Animal Tissue DNA Extraction Kit (Tiangen, Beijing, China). MethylRAD libraries were prepared for each sample according to the method described by Wang et al. [22], with FspEI (NEB, Ipswich, MA, USA) used for genomic DNA digestion. After library preparation, the libraries were pooled in equal amounts and sequenced on an Illumina NextSeq 500 platform (San Diego, CA, USA) using 150 bp paired-end sequencing. The raw data were submitted to NCBI SRA under accession number PRJNA890278.

### 2.3. Methylation Data Analysis

Raw reads were first trimmed by in-house python script to remove those with over 10% of low-quality base pairs and those lacking the FspEI recognition site [23]. Then, the filtered fragments were mapped to *Ancherythroculter nigrocauda* genome with Bowtie2 v2.3.5.1 [24]. After mapping, read counts at individual FspEI-digested methylation loci were used as a measure of relative methylation abundance. The methylation level for each CpG site was calculated according to the method in Wang et al. [22]. Loci were retained for differential methylation analysis when the total read count across all samples was ≥10 and the counts per million (CPM) exceeded 1 in at least three samples. Differential methylation between females and males was analyzed using the DESeq2 package in R, with females specified as the reference group [25,26]. Raw read counts were normalized using the size-factor normalization implemented in DESeq2, and differential methylation was assessed using the negative binomial model and Wald test. *p* values were adjusted for multiple testing using the Benjamini–Hochberg false discovery rate procedure, and loci with an adjusted *p* value < 0.05 were considered differentially methylated sites (DMSs). No fold-change cutoff was applied for DMS identification. Genes with more than 3 DMSs in the 2 kb upstream of transcript start site were kept as differential methylated genes in this study [27]. The position of methylated cytosine was placed with corresponding gene by bedtools v2.27.1 [28].

Before GO enrichment analysis, a custom annotation database (OrgDb v1.0) was constructed from eggNOG-mapper (2.1.12) [29] and Interproscan (5.72–103.0) [30] annotations. In detail, functional annotation was performed with eggNOG-mapper (v2.1.12) using DIAMOND and Actinopterygii taxonomic scope. Where available, InterProScan GO terms were merged with eggNOG-derived GO mappings to improve annotation coverage. Gene-level GO mappings were constructed by collapsing transcript/protein isoforms to gene identifiers and removing GO root terms. A custom Bioconductor OrgDb package (OrgDb v1.0) was built using AnnotationForge to support GO enrichment. For KEGG-related analyses, KO/pathway/module annotations derived from eggNOG were analyzed using TERM2GENE-based enrichment in clusterProfiler [31].

### 2.4. Chromosomal Distribution and Regional Analysis of Sex-Biased Methylation

To characterize the chromosomal distribution of sex-associated methylation differences, the reference genome was divided into non-overlapping 250 kb bins. The numbers of male-hypermethylated DMSs, female-hypermethylated DMSs, and predicted FspEI recognition motifs were determined for each bin. Bins containing fewer than 20 predicted FspEI motifs were excluded from further analysis. For each retained bin, a 2 × 2 contingency table was constructed using the numbers of female-hypermethylated and male-hypermethylated DMSs and their respective differences from the total motif count. Differences in the relative representation of female- and male-hypermethylated DMSs were assessed using a two-sided Fisher’s exact test. The resulting *p* values were adjusted across bins using the Benjamini–Hochberg procedure, and bins with adjusted *p* < 0.05 were classified as significantly sex-biased. The direction of bias was assigned according to whether the number of female-hypermethylated or male-hypermethylated DMSs was greater. The magnitude of directional bias was summarized as log_2_[(f + 0.5)/(m + 0.5)], where f and m represent the corresponding DMS counts.

For each superscaffold, we calculated the proportion of significant bins, the longest consecutive run of significant bins, and the proportion of significant bins sharing the predominant direction of methylation bias. These measures were combined into an exploratory scaffold-ranking score defined as 5 × fraction of significant bins + 0.5 × longest significant-bin run + 2 × directional consistency. Scaffolds with high scores were prioritized for further investigation of extended sex-associated methylation patterns.

### 2.5. Correlation Analysis Between Methylation and RNA-Seq Expression Level

Because the methylation and transcriptomic datasets were derived from the same biological individuals, direct integration of DNA methylation and gene expression profiles minimized inter-individual variation between the two datasets. The gene expression data of liver tissue were retrieved from our previous article [9]. Genes were stratified into biologically informed categories according to DMS burden (0, 1, 2–3, 4–6, 7–10, 11–20, and >20 sites), and enrichment of DEGs within each bin was calculated relative to the genome-wide background. To assess the effect of extreme methylation, genes harboring > 20 DMS were compared with all remaining genes using Fisher’s exact test, with odds ratios and 95% confidence intervals reported. Sex-skewed methylation was defined as a difference of at least three DMS between sexes with a minimum of 3 sites in the dominant sex. Directional concordance between methylation bias and sex-biased expression was evaluated by overlap analysis and tested using Fisher’s exact test. Statistical analyses and visualization were performed in R 4.4 (R Core Team, Vienna, Austria) using the *ggplot2* package.

## 3. Results

### 3.1. Genome Architecture and Genome-Wide Distribution of Symmetric FspEI Methylation Targets

To establish the genomic framework for MethylRAD profiling, we first evaluated the distribution of potential FspEI recognition sites across the genome of *Ancherythroculter nigrocauda*. FspEI cleaves double-stranded DNA on the 3′ side of methylated cytosines at a fixed distance (N12/N16), generating canonical 32 bp fragments when target sites are symmetrically methylated [22]. This predictable cleavage pattern enables robust identification of methylated loci and makes the genomic architecture of recognition motifs a critical determinant of methylome coverage.

**Figure 1:**
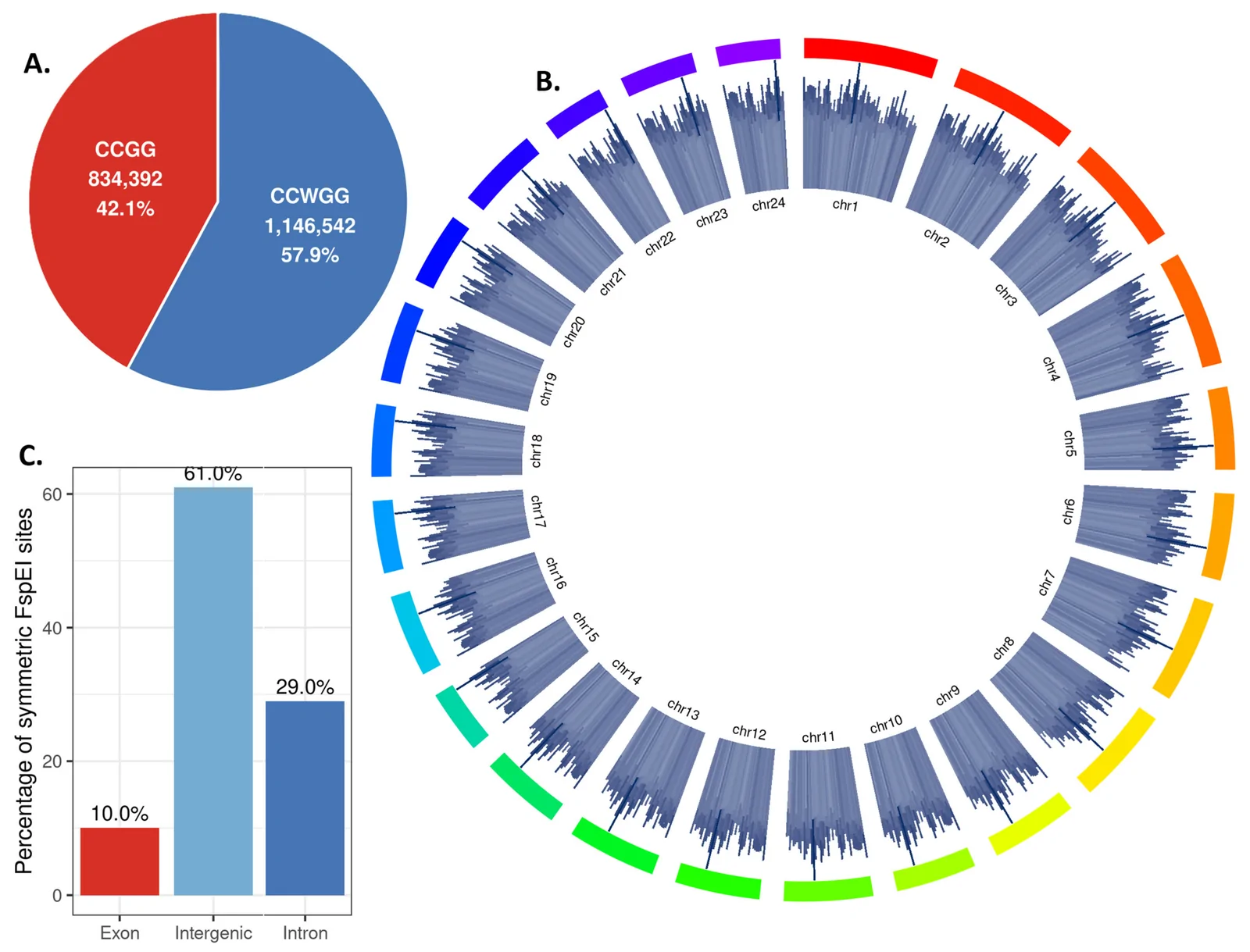
Genome-wide in silico evaluation and distribution of FspEI recognition sites. (**A**) Proportion of symmetric FspEI recognition motifs identified in the genome. (**B**) Circos plot showing the chromosomal distribution and density of symmetric FspEI sites across all chromosomes. The colored segments in the outer ring represent individual chromosomes, with segment length proportional to chromosome size; different colors are used solely to visually distinguish adjacent chromosomes and do not represent additional biological information. The inner histogram shows the density of symmetric FspEI recognition sites along each chromosome. (**C**) Genomic annotation of symmetric FspEI sites relative to gene features.

In silico digestion identified 81,341,472 potential FspEI recognition sites, among which 1,980,934 (2.44%) were classified as primary symmetric fragments (CCGG and CC(A/T)GG). Because these loci produce canonical fragments only when methylated on both strands, they represent high-confidence targets for methylation detection. Of these, 834,392 were CCGG motifs and 1,146,542 were CCWGG motifs, with average inter-site distances of 1263 bp and 919 bp, respectively (Figure 1A). The greater abundance of CCWGG motifs suggests that non-CG methylation contexts may substantially contribute to the epigenetic landscape of this species, consistent with emerging evidence that CHG methylation participates in vertebrate gene regulation.

Having defined the motif repertoire, we next examined their chromosomal distribution to determine whether MethylRAD provides balanced genome representation. Symmetric motifs were broadly dispersed across all chromosomes with no evident clustering, indicating uniform sampling of genomic space (Figure 1B). Functional annotation further showed that these motifs occur throughout promoter, exonic, intronic, and intergenic regions, demonstrating that FspEI targets span both regulatory and structural genomic elements (Figure 1C). Notably, approximately 13% of motifs were located within promoter regions (±2 kb of transcription start sites), representing enrichment relative to the genomic proportion of promoter space and suggesting strong coverage of transcriptional regulatory regions.

Because regulatory DNA is frequently associated with CpG-rich sequences, we next investigated the relationship between FspEI motifs and CpG island architecture. A total of 79,513 CpG islands were identified, spanning 26.66 Mb and accounting for approximately 2.53% of the genome (Supplementary Figure S1A). These islands were relatively short (mean 335 bp) and widely dispersed, consistent with the CpG organization typical of teleost genomes [30,31]. Only 14.6% were located within ±2 kb of transcription start sites, supporting previous observations that teleost CpG islands are less promoter-concentrated than those of mammals (Supplementary Figure S1B).

Importantly, symmetric FspEI recognition sites showed strong preferential localization within CpG islands. A total of 208,468 sites (9.9%) overlapped CpG islands—nearly a four-fold enrichment compared with random genomic expectation—indicating that FspEI-sensitive loci are closely associated with regulatory DNA (Supplementary Figure S1A). Furthermore, CpG-containing CCGG motifs and non-CpG CCWGG motifs exhibited nearly identical promoter representation (~13%), suggesting minimal motif-specific bias and supporting the capacity of MethylRAD to interrogate both CpG and non-CpG methylation contexts without systematic positional skew.

### 3.2. Sequencing Output and Symmetric FspEI Methylation Target Detection

MethylRAD sequencing generated high-quality datasets across all 5 female and 5 male *Ancherythroculter nigrocauda* samples, with raw read counts ranging from 31.1 million to 57.4 million per library (Table 1). After quality filtering, 97.1–98.2% of reads were retained, indicating minimal adapter contamination and overall excellent library construction. Alignment to the reference genome produced consistently high mapping efficiencies, with mapping rates between 89.7% and 94.0%. Among mapped reads, the proportion of uniquely aligned reads ranged from 42.3% to 49.5%, suggesting reliable genomic placement despite the reduced-representation nature of MethylRAD libraries.

Using the methylation-dependent restriction enzyme FspEI, two motif types were profiled: CCGG and CCWGG. Across samples, substantially more methylated loci were detected at CCGG sites than at CCWGG sites. The number of CCGG-derived reads ranged from 4.87 million to 11.44 million, whereas CCWGG-derived reads ranged from 1.15 million to 2.70 million. Although absolute counts varied among samples, the ratio between CCGG and CCWGG reads remained relatively stable, supporting consistent digestion efficiency and reproducible methylation capture across libraries.

After merging overlapping reads, each library captured approximately 1.03–1.47 million unique FspEI digested loci, demonstrating consistent genome representation across samples. The comparable numbers of detected loci suggest that sequencing depth was sufficient to achieve stable genome-wide coverage, providing a reliable foundation for subsequent comparative methylation analyses.

### 3.3. Genome-Wide Identification and Regulatory Context of Sex-Biased DNA Methylation

**Figure 2:**
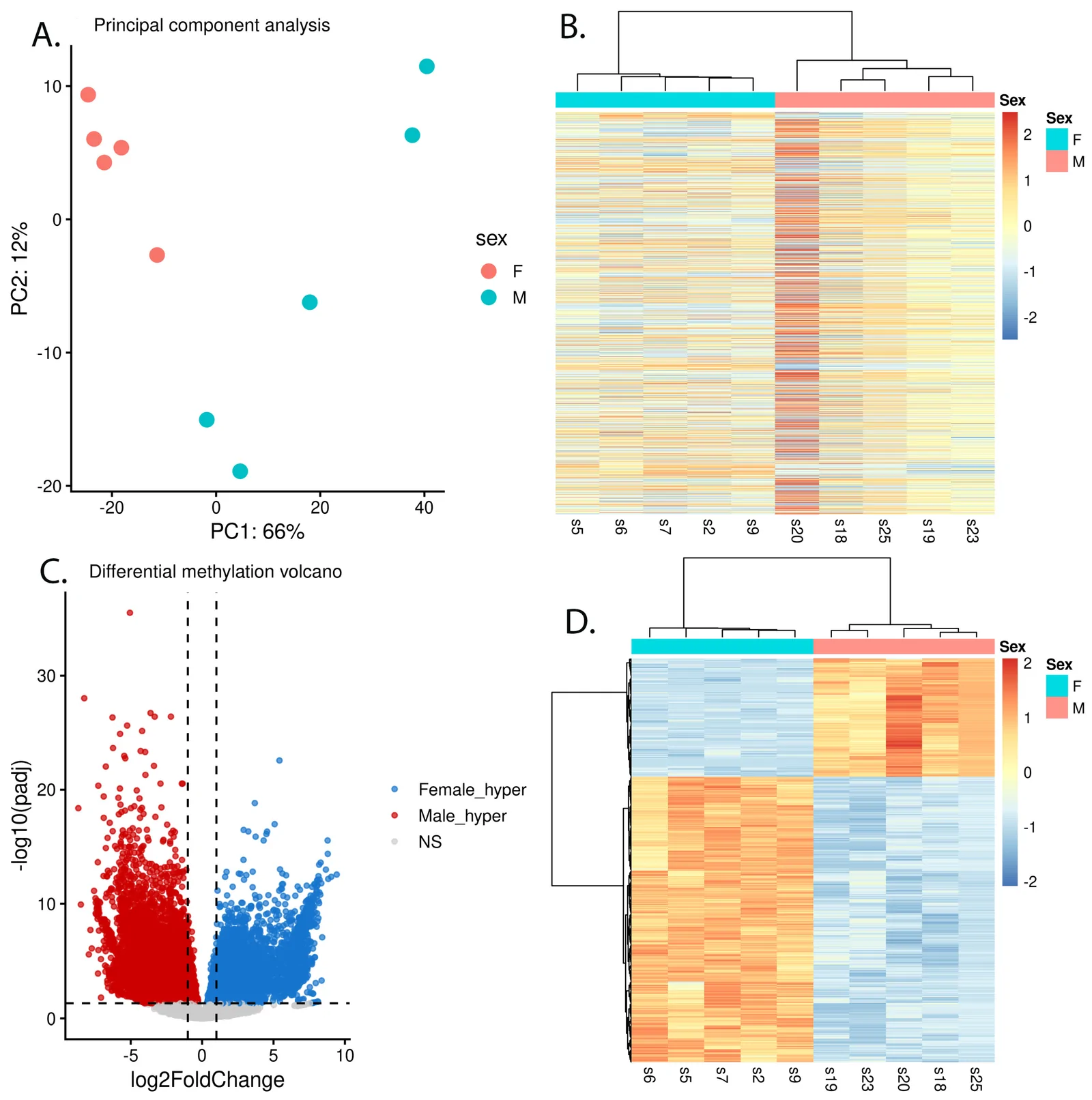
Genome-wide identification of sex-biased DNA methylation. (**A**) Principal component analysis showing separation of male and female samples. (**B**) Hierarchical clustering of loci with adjusted *p* < 0.01. (**C**) Volcano plot illustrating differentially methylated loci between sexes. Vertical dashed lines are log_2_ fold-change thresholds of −1 and +1, corresponding to an absolute fold change of 2. Horizontal dashed line is adjusted *p*-value threshold of 0.05. (**D**) Heatmap of the top 1000 differentially methylated loci highlighting reproducible sex-specific patterns.

To investigate sex-associated epigenetic variation, genome-wide methylation profiles were compared between male and female groups. Principal component analysis (PCA) based on variance-stabilized methylation levels revealed clear separation of samples by sex, indicating distinct global methylation signatures (Figure 2A). Hierarchical clustering of loci exhibiting strong statistical support (adjusted *p* < 0.01) further segregated male and female samples into discrete groups, demonstrating high within-group consistency and suggesting robust sex-specific methylation patterns (Figure 2B).

A total of 973,937 loci were tested, resulting in the identification of 82,872 differentially methylated sites (DMSs) under the defined statistical thresholds (*p*adj < 0.05, Figure 2C, Supplementary Table S1). Among these, 35,903 sites (43.32%) were hypermethylated in males, whereas 46,968 (56.67%) were hypermethylated in females, indicating substantial epigenetic divergence between the sexes. Visualization using a volcano plot showed symmetric distributions of male- and female-biased loci, indicating that sex-associated methylation changes occurred in both directions rather than being dominated by a single global shift (Figure 2C). To highlight the most prominent epigenetic differences, the top 1000 differentially methylated loci were subjected to unsupervised clustering. These loci produced a clear dichotomy between male and female samples, emphasizing the reproducibility and biological relevance of sex-biased methylation signals (Figure 2D).

To further investigate the biological relevance of gene-body methylation, we focused on the top 20 genes with the largest number of differentially methylated sites (DMSs) in females and males. In females, highly methylated genes were predominantly associated with regulatory and immune-related functions. Notably, tripartite motif-containing protein 47 (ftr61/TRIM47-like) exhibited substantial methylation density, alongside multiple transcriptional regulators such as zinc finger proteins. Additionally, protein tyrosine phosphatase receptor type D (PTPRD)—a key mediator of cell adhesion and intracellular signaling—was among the most heavily methylated genes, suggesting sex-specific epigenetic modulation of signaling pathways.

In contrast, the male top-DMS genes displayed a narrower functional profile, with strong representation of innate immune effectors. The most prominent example was complement component C3 (c3), a central regulator of the complement cascade and pathogen clearance. While PTPRD was also detected among highly methylated genes in males, we found male and female DMSs were evenly distributed on the whole-genome region for male and female fish (Supplementary Figure S2).

To evaluate whether differentially methylated sites (DMSs) were preferentially distributed within transcription start site (TSS)-proximal regions and CpG islands, we performed enrichment analyses using all 342,699 loci included in the DESeq2 analysis as the background. Among the 82,871 significant DMSs, 10,667 (12.87%) overlapped TSS-proximal regions (±2 kb), compared with 45,592 (13.30%) of all tested loci. Fisher’s exact test revealed a modest but statistically significant depletion of DMSs in these regions (fold enrichment = 0.968; odds ratio = 0.951; adjusted *p* = 2.54 × 10^−5^). Similarly, 9750 DMSs (11.77%) overlapped CpG islands, compared with 42,180 (12.31%) of all tested loci, indicating a modest depletion (fold enrichment = 0.956; odds ratio = 0.935; adjusted *p* = 8.36 × 10^−8^). These results indicate that sex-associated methylation differences were not preferentially enriched in TSS-proximal regions or CpG islands after accounting for the genomic distribution of tested MethylRAD loci.

**Figure 3:**
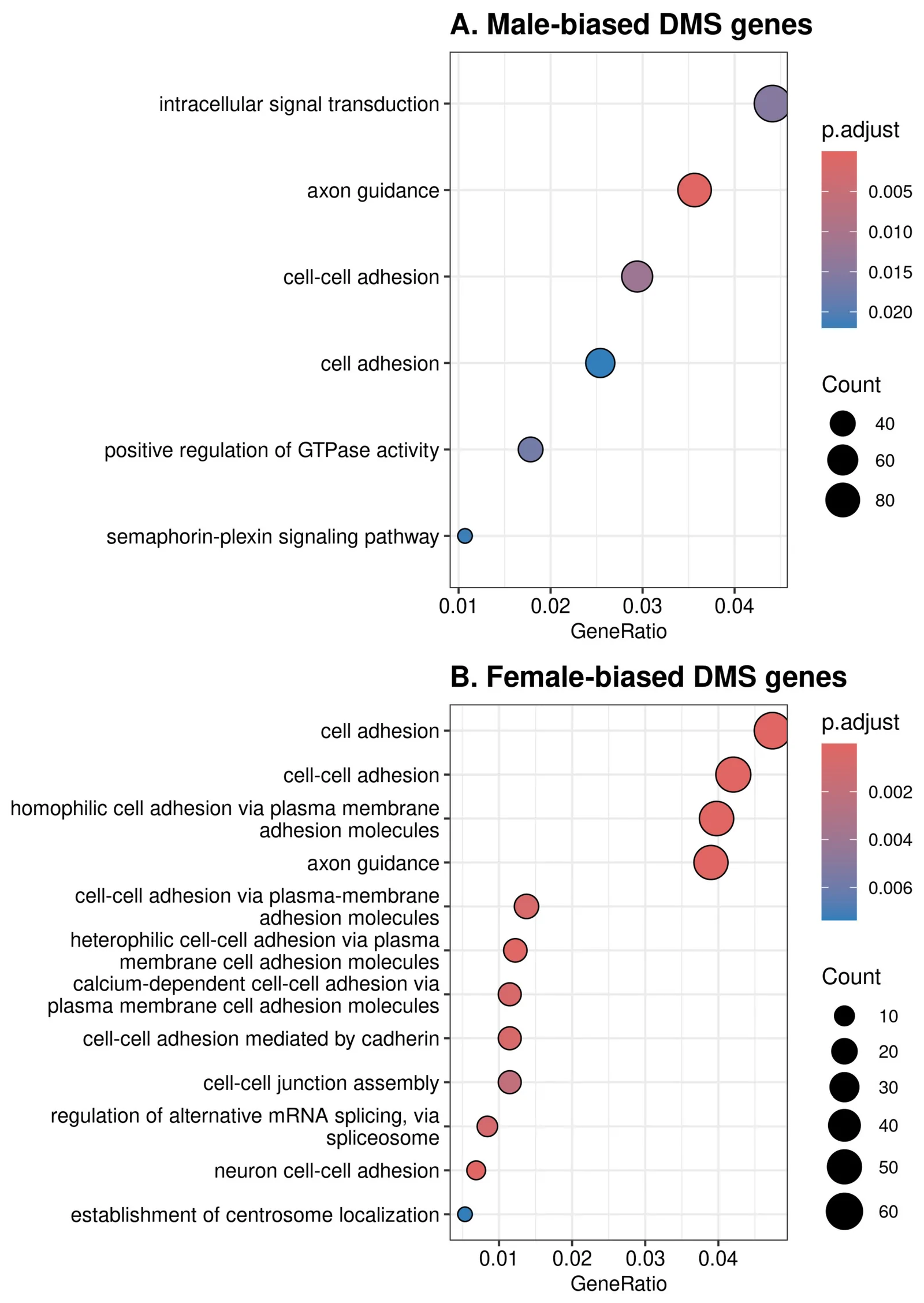
Sex-dependent functional divergence of differentially methylated genes in liver. Gene Ontology Biological Process enrichment was conducted for genes harboring > 3 differentially methylated sites. Dotplots illustrate significantly enriched pathways (FDR < 0.05), where dot size corresponds to gene count and color reflects adjusted significance. (**A**) Male-biased methylation was enriched in pathways governing cell adhesion and developmental signaling, indicating enhanced regulation of intercellular communication and tissue architecture. (**B**) Female-biased methylation preferentially targeted genes involved in RNA processing, epithelial polarity, and apoptosis, consistent with increased transcriptional plasticity and tissue remodeling associated with larger liver mass in females.

Then, we filtered genes with at least four DMSs for the following enrichment analysis; among them, 3248 were male-hypermethylated and 1978 were female-hypermethylated out of the 19,623 background gene set, which has at least one DMS in it. Male-biased DMS genes showed significant enrichment for biological processes primarily related to intracellular signaling and cell communication (Figure 3A). The most enriched category was intracellular signal transduction, followed by pathways associated with axon guidance, cell adhesion, and cell–cell adhesion. Additionally, enrichment of positive regulation of GTPase activity and the semaphorin–plexin signaling pathway suggests enhanced regulation of cytoskeletal dynamics and signal-mediated cellular responses in male liver tissue.

Female-biased DMS genes exhibited a distinct enrichment profile dominated by processes involved in cell adhesion and intercellular junction organization (Figure 3B). Highly significant terms included *cell adhesion*, *cell–cell adhesion*, and multiple adhesion-related mechanisms mediated by plasma membrane molecules and cadherins. Enrichment of *axon guidance* was also observed, indicating shared regulatory pathways between the sexes but potentially differing epigenetic targeting. Notably, processes related to *cell–cell junction assembly* and *calcium-dependent adhesion* suggest tighter regulation of tissue architecture in females. Additional enrichment for *regulation of alternative mRNA splicing via the spliceosome* points toward sex-specific epigenetic modulation of post-transcriptional gene regulation.

### 3.4. Chromosomal Distribution of Differentially Methylated Sites Reveals Candidate Sex-Linked Regions

To investigate the genomic landscape of sex-biased DNA methylation, differentially methylated sites (DMSs) identified by DESeq2 (adjusted *p* < 0.05) were aggregated into 250 kb non-overlapping genomic bins. A total of 776 bins exhibited significant sex-biased methylation across the genome. Among these, 547 bins (70.5%) were male-biased, whereas 229 bins (29.5%) were female-biased, indicating a pronounced genome-wide tendency toward higher methylation levels in males. A binomial test confirmed that this skew significantly deviated from the null expectation of equal distribution between sexes (*p* < 0.001), suggesting that male hypermethylation represents a global epigenetic feature in this species.

Because methylRAD signals may be influenced by restriction enzyme site density, we evaluated the relationship between motif abundance and DMS counts. Motif density showed only a weak correlation with DMS number (Pearson’s *r* = 0.287), indicating that the observed sex bias is unlikely to be driven primarily by technical variation and instead reflects genuine biological differences.

**Figure 4:**
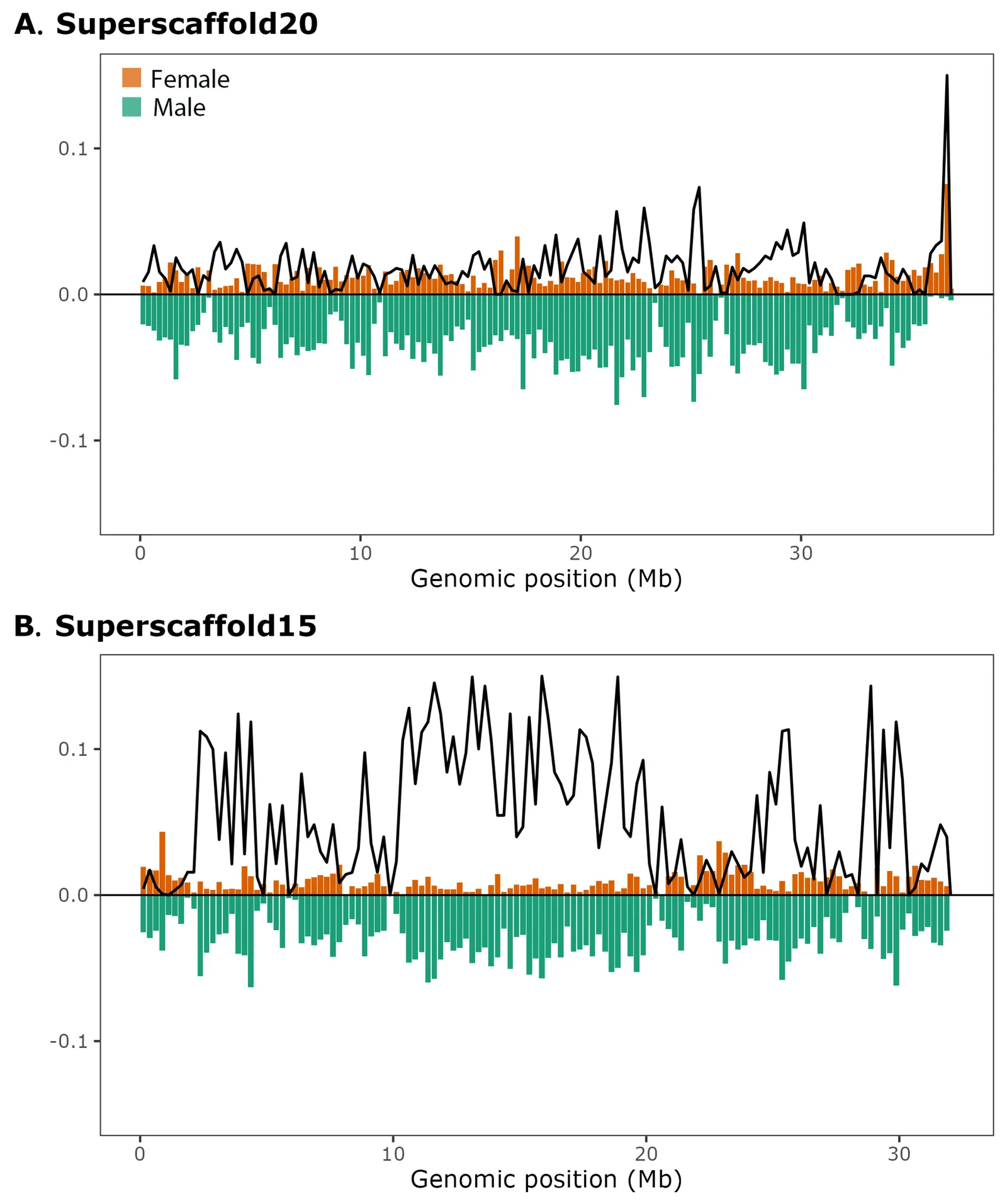
Regional landscapes of sex-biased differentially methylated sites on candidate sex-linked scaffolds. (**A**) Superscaffold20 and (**B**) Superscaffold15 display the genomic distribution of sex-biased differentially methylated sites (DMS) aggregated into 250 kb non-overlapping bins. Orange bars above the horizontal axis represent female-hypermethylated DMS density, whereas green bars below the axis indicate male-hypermethylated DMS density. The black line denotes the relative significance of sex bias for each bin (−log_10_ adjusted *p*-value, scaled to panel height).

Notably, the distribution of significant bins was highly non-random across chromosomes. Several superscaffolds exhibited substantial enrichment, with Superscaffold15 showing the strongest signal: 54 of 129 bins (41.9%) were significantly male-biased, and no female-biased bins were detected on this scaffold (Figure 4, sexbias_bins_250kb.tsv). The regional distributions of female- and male-hypermethylated DMSs are visualized in Figure 4 as orange and green bars, respectively, revealing pronounced spatial clustering of sex-biased methylation along these scaffolds. In addition, extended contiguous regions of sex-biased methylation were observed, including two large blocks spanning 15 and 11 consecutive bins (approximately 2.8–3.8 Mb each, Supplementary Table S2). Such long, directionally consistent methylation domains are characteristic of recombination-suppressed regions and are frequently associated with emerging sex chromosomes in teleost fishes. Functional annotation of genes located within the differentially methylated region on Superscaffold15 identified multiple candidates associated with gonadal development and reproductive biology. Notably, the region contains *wt1*, a highly conserved transcription factor essential for bipotential gonad formation and testis differentiation across vertebrates. The presence of *kitlg*, which regulates primordial germ cell survival and gametogenesis, further supports the biological relevance of this locus. Additional genes involved in developmental signaling (e.g., *met*, *fgf6*), steroid metabolism (cytochrome P450 family members), and epigenetic regulation were also detected. The enrichment of genes associated with reproductive development suggests that Superscaffold15 likely represents a candidate sex determination region in this species.

Superscaffold20 also displayed strong enrichment (57 of 148 bins; 38.5%), predominantly male-biased (53 male vs. 4 female). A continuous 2.25 Mb segment (28.00–30.25 Mb) showed strong male-biased methylation, whereas a separate ~1 Mb interval (35.75–36.75 Mb) displayed female-biased methylation (Supplementary Table S2). Notably, our previous genomic investigation detected a region (31.60–32.50 Mb) of sex-specific sequencing coverage located between these intervals [32]. This coverage-differentiated region is located between the two methylation blocks but does not overlap either of them. Thus, the sex-biased methylation blocks flank the previously identified coverage-differentiated region on Superscaffold20, highlighting this scaffold as a candidate sex-linked genomic region for further investigation.

Although additional scaffolds showed moderate enrichment, none matched the combination of high fraction of significant bins, strong directional consistency, and large contiguous blocks observed for Superscaffold15 and Superscaffold20. Together, these results suggest that sex-biased methylation is not uniformly distributed but instead concentrates within discrete genomic regions, consistent with the early evolution of sex chromosomes or recombination-suppressed domains.

Overall, the genome-wide methylation patterns identify Superscaffold15 and Superscaffold20 as candidate regions exhibiting pronounced sex-associated methylation. On Superscaffold20, the proximity of these methylation blocks to the previously identified, non-overlapping coverage-differentiated region provides additional motivation for further investigation of this scaffold. However, the present methylation data do not establish either scaffold as a primary sex determination locus. Further genomic and functional analyses will be required to determine whether these regions are associated with sex determination, sex linkage, or other sex-specific regulatory processes. To evaluate whether differential methylation directly targets conserved components of the vertebrate sex determination pathway, we manually examined gene-level methylation patterns for canonical regulators, including *sox9*, *foxl2*, *wt1*, *sf1* (nr5a1), *nr0b1*, *gata4*, and *dhh*. These genes were all identifiable within the genome annotation, confirming high annotation completeness for sex-related pathways in this species. Overall, differential methylation across these loci was limited, with most genes exhibiting few or no sex-biased methylation sites in liver tissue (Supplementary Table S3). Nevertheless, modest male-specific methylation was detected at *wt1* and *gata4*, while one *sox9* paralog displayed a female-biased methylation signal. The generally sparse methylation differences observed in these master regulators suggest that sex-specific epigenetic regulation is unlikely to be maintained broadly in somatic tissues. Instead, these results are consistent with a regulatory model in which epigenetic divergence between sexes is either tissue-specific—potentially concentrated in gonadal tissue—or confined to earlier developmental stages when sex fate is established.

### 3.5. Gene Ontology Enrichment Reveals Sex-Biased Regulatory Pathways Associated with Hepatic Growth

Gene set enrichment analysis (GSEA) based on genome-wide methylation bias identified multiple significantly enriched biological processes (adjusted *p* < 0.05), indicating coordinated epigenetic differences between sexes rather than gene-specific effects. Several enriched terms were linked to intracellular signaling and protein modification, including activation of GTPase activity, peptidyl-serine phosphorylation, and peptidyl-serine modification. These pathways are central regulators of cellular proliferation, metabolic activity, and growth-related signal transduction, suggesting that sex-biased methylation may influence hepatic growth dynamics through modulation of downstream signaling networks.

**Figure 5:**
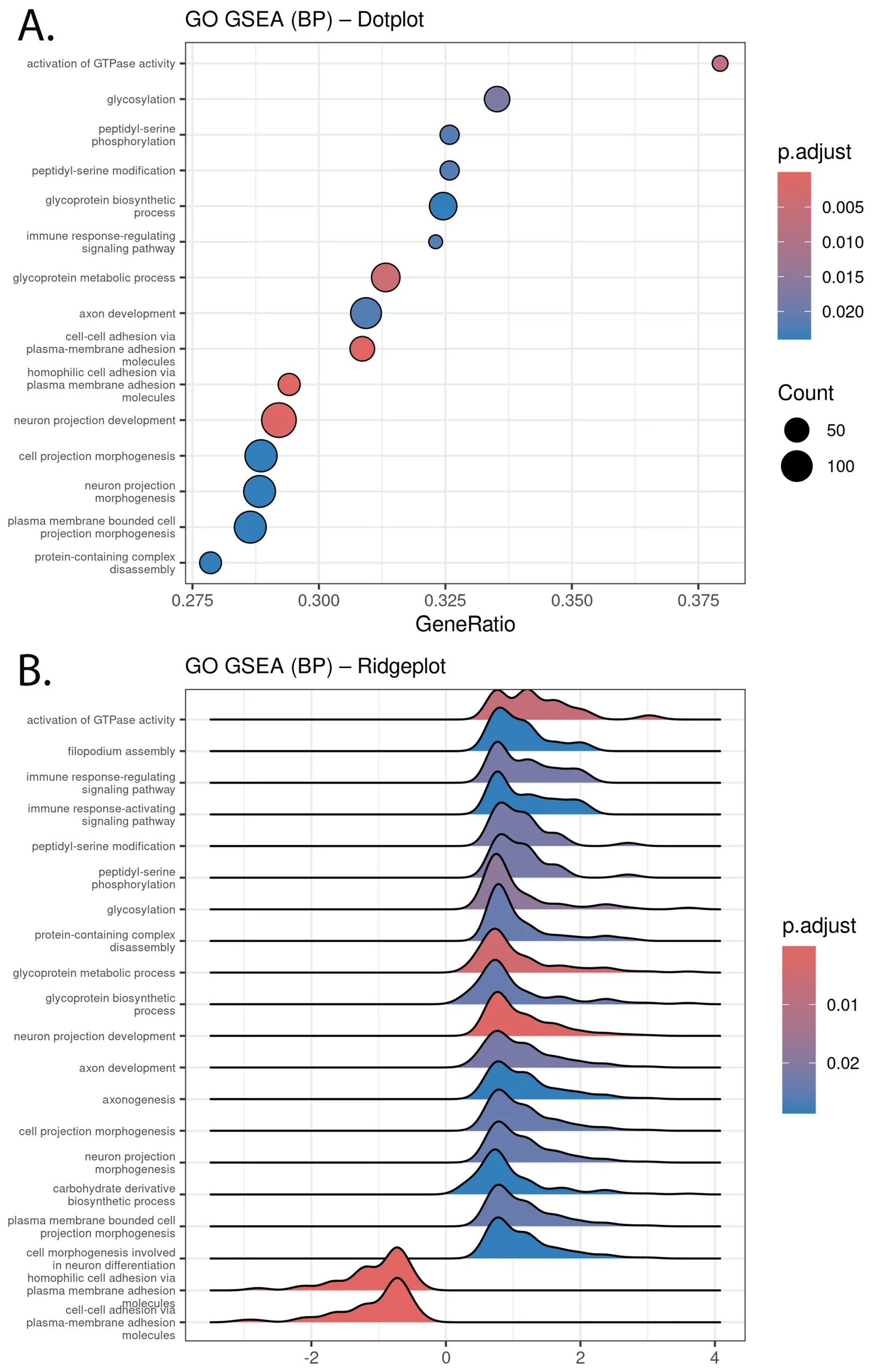
Gene Ontology enrichment analysis of sex-biased DNA methylation in liver. Gene set enrichment analysis (GSEA) was performed using genome-wide gene rankings based on log_2_-transformed methylation differences between females and males, weighted by methylation site abundance. (**A**) Dotplot showing significantly enriched biological processes (adjusted *p* < 0.05). Dot size represents the number of genes contributing to each pathway, and color indicates the adjusted *p*-value. (**B**) Ridgeplot illustrating the distribution of enrichment signals across ranked genes for selected pathways. Positive enrichment scores indicate pathways associated with relatively higher methylation in one sex, whereas negative scores reflect enrichment toward the opposite sex. Enriched processes were primarily associated with intracellular signaling, protein modification, glycoprotein metabolism, and cellular morphogenesis, suggesting coordinated epigenetic regulation of pathways relevant to sexually dimorphic liver function and growth.

Processes associated with protein glycosylation and glycoprotein metabolism were also prominently enriched. Glycosylation plays essential roles in hormone receptor function, protein stability, and nutrient metabolism, all of which are critical determinants of liver growth and physiological performance in teleost fishes. Differential methylation within these pathways, therefore, points to potential sex-specific regulation of metabolic capacity. Notably, several enriched categories were related to cell morphogenesis and structural organization, including cell projection morphogenesis, axon development, and neuron projection morphogenesis. Although traditionally associated with neural biology, these processes also reflect cytoskeletal remodeling and cellular architecture, which are fundamental for hepatocyte growth and tissue expansion. Additionally, enrichment of cell adhesion pathways, such as homophilic cell adhesion via plasma membrane adhesion molecules and cell–cell adhesion, suggests possible sex-dependent variation in hepatic tissue organization. Because cell adhesion influences both organ structure and regenerative capacity, these findings further support a role for epigenetic regulation in shaping growth-related liver phenotypes (Figure 5A).

The ridgeplot further demonstrated pronounced directional enrichment across ranked genes, indicating that sex-biased methylation is broadly distributed throughout the genome rather than confined to isolated loci. The clustering of gene density toward enrichment peaks suggests coordinated epigenetic regulation across functionally related gene sets, consistent with a polygenic architecture underlying sexually dimorphic liver biology. Such genome-wide shifts are more indicative of regulatory reprogramming than localized methylation events and provide additional support for the role of epigenetic mechanisms in shaping sex-specific growth-related pathways. Together with the dotplot results, the ridgeplot provides complementary evidence that sex-specific methylation preferentially targets coordinated biological pathways (Figure 5B).

Collectively, these results indicate that sex-biased DNA methylation preferentially targets regulatory pathways governing signal transduction, protein metabolism, and cellular architecture, highlighting a potential epigenetic basis for sexually dimorphic growth regulation in the liver. These patterns are consistent with the hypothesis that epigenetic regulation contributes to sexually dimorphic growth observed in this species. Together, these findings suggest that sex-biased methylation may represent an important regulatory mechanism underlying the hepatic component of sexually dimorphic growth.

### 3.6. Integration of Differential Methylation and Transcription Reveals a Threshold-Dependent Regulatory Architecture

To investigate the relationship between DNA methylation and sex-biased transcription in the liver, we integrated gene-level differential methylation with our previous liver RNA-seq differential expression profiles [9]. Genome-wide assessment revealed a highly skewed distribution of differentially methylated sites (DMSs) across genes. Over half of the annotated genes lacked detectable methylation differences, whereas a small subset exhibited substantial methylation clustering, with some genes harboring more than 200 DMSs. This distribution suggests that sex-associated methylation is concentrated within a restricted portion of the genome rather than broadly dispersed.

**Figure 6:**
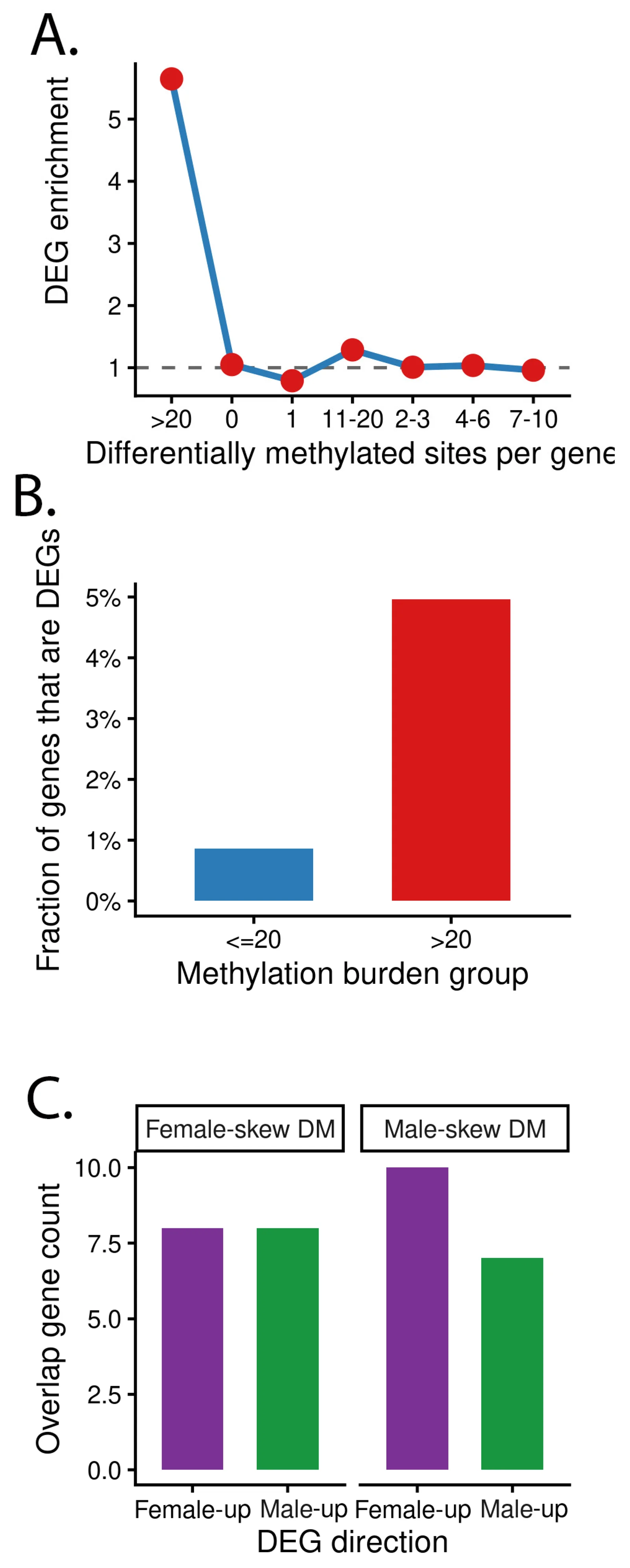
Extreme methylation burden and sex-skewed methylation are associated with sex-biased gene expression in liver. (**A**) Genes were stratified by the number of differentially methylated sites (DMS) per gene to evaluate the relationship between methylation burden and transcriptional divergence. DEG enrichment represents the relative enrichment of differentially expressed genes within each DMS-burden category. The dashed horizontal line indicates an enrichment value of 1 (no enrichment). (**B**) Threshold analysis confirmed this pattern: genes with >20 DMS displayed a nearly six-fold higher probability of differential expression compared with genes possessing ≤ 20 DMS (odds ratio = 5.99, Fisher’s exact test *p* = 2.6 × 10^−4^). Blue represents genes with ≤20 DMS, whereas red represents genes with >20 DMS. (**C**) Association between the direction of sex-skewed methylation and sex-biased gene expression. Purple bars represent female-upregulated differentially expressed genes, whereas green bars represent male-upregulated genes. The two panels show genes with female-skewed and male-skewed methylation, respectively. Fisher’s exact test did not identify a significant association between methylation direction and expression direction (two-sided *p* ≈ 0.73).

We next evaluated whether methylation burden was associated with transcriptional divergence. Genes were stratified according to the number of differentially methylated sites (DMSs) per gene, revealing minimal enrichment for differential expression among genes with low to moderate methylation levels but a sharp increase among heavily methylated loci (Figure 6A). In contrast, genes containing more than 20 DMSs displayed a marked increase in the likelihood of sex-biased expression. Specifically, 4.96% of highly methylated genes were differentially expressed compared with 0.86% of genes with lower methylation burden, representing an approximately 6-fold enrichment (odds ratio = 5.99, 95% CI = 2.34–12.80; Fisher’s exact test *p* = 2.6 × 10^−4^; Figure 6B). These findings indicate the presence of a threshold-dependent relationship, in which extreme methylation clustering, rather than incremental increases in methylation, is preferentially associated with transcriptional divergence between sexes.

To further investigate the relationship between sex-skewed methylation and sex-biased transcription, we examined the directional distribution of differentially expressed genes associated with female- and male-skewed methylation (Figure 6C). Among genes exhibiting female-skewed methylation, eight were upregulated in females and eight in males. Among genes exhibiting male-skewed methylation, ten were upregulated in females and seven in males. Fisher’s exact test revealed no significant association between the direction of methylation bias and the direction of differential expression (two-sided *p* ≈ 0.73). Thus, although genes with a high DMS burden were more likely to exhibit differential expression (Figure 6B), the direction of methylation bias did not consistently predict the direction of transcriptional change.

Collectively, these results suggest that epigenetic regulation of liver gene expression is not governed by diffuse genome-wide methylation differences but is instead concentrated within a limited subset of highly methylated genes. Such loci may represent regulatory hotspots that contribute disproportionately to the establishment of sex-specific transcriptional programs.

## 4. Discussion

Sexual dimorphism is widespread in fishes and commonly manifests as differences in coloration, body size, or growth rate between sexes [33]. Despite its prevalence, the genetic and epigenetic mechanisms underlying sexual size dimorphism (SSD) remain insufficiently understood, particularly in non-model species [34]. DNA methylation is a major regulatory mechanism influencing gene expression and developmental processes, making genome-scale characterization of methylation landscapes critical for understanding the molecular basis of SSD. Reduced-representation methylation approaches such as MethylRAD provide a cost-effective strategy for profiling genome-wide DNA methylation at regulatory loci, offering new opportunities to investigate epigenetic contributions to sex-biased traits [22].

In this study, we used MethylRAD to study the epigenetic difference between female and male liver tissue for *Ancherythroculter nigrocauda*, a fish with female-biased SSD. MethylRAD had been used in many epigenetic studies, which aimed at finding methylation differences between various phenotypes [26,35]. It has many advantages compared with whole-genome bisulfite sequencing or reduced represented bisulfite sequencing, not needing to convert ^m^C to T before library construction. And it only needs one gel purification during the sequencing library construction [22]. In the *Ancherythroculter nigrocauda* genome, the genomic architecture of symmetric FspEI recognition sites revealed broad genome representation with enrichment in promoter regions and CpG islands, indicating that MethylRAD effectively captures functionally relevant regulatory elements. The substantial presence of both CpG and non-CpG motifs further suggests that multiple methylation contexts may contribute to transcriptional regulation in this species. Given the relatively dispersed CpG island organization typical of teleost genomes, such coverage is particularly important for detecting regulatory variation beyond canonical promoter regions.

The present study revealed extensive sex-biased DNA methylation in the liver of *Ancherythroculter nigrocauda*, with more than 80,000 DMSs distinguishing male and female methylation profiles. After accounting for the distribution of tested MethylRAD loci, DMSs showed modest but statistically significant depletion in both TSS-proximal regions (±2 kb) and CpG islands. These findings suggest that sex-associated methylation differences are not preferentially concentrated within these regulatory features but occur across multiple genomic contexts accessible to MethylRAD. Although methylation within promoter-associated regions and CpG islands may influence transcriptional activity, the functional significance of individual DMSs cannot be inferred from genomic localization alone. Similar sex-associated methylation differences have been reported in other teleost species, including Nile tilapia (*Oreochromis niloticus*) and half-smooth tongue sole (*Cynoglossus semilaevis*) [36,37]. In our liver dataset, genes associated with sex-biased methylation showed functional enrichment in intracellular signaling, cell adhesion, axon guidance, and GTPase-mediated pathways, suggesting potential relationships with cellular communication, tissue organization, and physiological regulation. These processes may be relevant to growth hormone (GH)–insulin-like growth factor (IGF) signaling, nutrient sensing, and metabolic coordination, which contribute to sexual size dimorphism in teleost fishes [38,39]. Thus, the potential regulatory significance of sex-biased methylation may depend more on the specific genes and biological pathways affected than on a general preference for promoter-associated or CpG-island regions.

Functional divergence between male- and female-biased DMS genes further supports a role for methylation in shaping dimorphic growth physiology. Female-biased methylation was associated with enhanced regulation of cell–cell adhesion, junction assembly, and spliceosomal pathways, implying tighter structural organization and post-transcriptional modulation of gene expression. In contrast, male-biased DMS genes showed enrichment in intracellular signaling and immune-related pathways, including complement component C3, highlighting potential differences in metabolic immune integration. In teleost livers, immune activity, energy allocation, and growth regulation are closely interconnected, and sex-dependent transcriptional or epigenetic regulation of these pathways has been documented in zebrafish and Atlantic salmon [40,41]. Together, our findings indicate that sex-biased DNA methylation in the liver is not merely a secondary effect of hormonal differences but likely acts as a regulatory layer modulating promoter activity, signaling cascades, and cellular architecture, thereby contributing to the molecular basis of sexual dimorphism in growth and metabolic performance in teleost fish.

The highly non-random chromosomal distribution of sex-biased methylation, particularly the large contiguous male-biased domains on Superscaffold15 and Superscaffold20, strongly suggests the presence of localized recombination-suppressed regions consistent with early-stage sex chromosome differentiation. In many teleost fishes, sex chromosomes are homomorphic or only partially differentiated, with sex-linked regions often restricted to discrete genomic blocks rather than entire chromosomes [39,42,43]. Extended domains of directionally consistent methylation spanning several megabases, as observed here, resemble epigenetic patterns described in emerging sex chromosomes of species such as Nile tilapia (*Oreochromis niloticus*) and medaka (*Oryzias latipes*), where recombination suppression is accompanied by regulatory divergence and chromatin modification [44,45]. The pronounced male-biased methylation enrichment within Superscaffold15 and Superscaffold20, therefore, supports the hypothesis that these regions represent candidate sex-linked loci undergoing structural or regulatory differentiation.

An important limitation of MethylRAD-based analyses is the potential confounding effect of underlying genetic variation. Because MethylRAD depends on methylation-sensitive cleavage at specific FspEI recognition sites, sequence polymorphisms that create or disrupt these motifs may alter fragment recovery independently of DNA methylation. Sex-linked sequence divergence, copy-number variation, and differences in read-mapping efficiency may, therefore, contribute to apparent sex-biased methylation signals. This possibility is particularly relevant to the extended regions identified on Superscaffold15 and Superscaffold20. On Superscaffold20, the methylation-biased blocks flank, but do not overlap, a previously identified interval exhibiting sex-specific sequencing coverage, suggesting that genetic variation should be considered when interpreting these patterns. Accordingly, the observed regional differences in MethylRAD signals cannot be attributed exclusively to epigenetic variation, and these scaffolds should be considered candidate regions requiring further investigation. Future studies incorporating whole-genome resequencing, analysis of sequence polymorphisms at FspEI recognition sites, copy-number assessment, and restriction-enzyme-independent methylation profiling will be necessary to distinguish genetic from epigenetic contributions. Comparative evidence from other teleosts further supports the coupling of sex-linked genomic regions with sex-specific methylation landscapes. In half-smooth tongue sole (*Cynoglossus semilaevis*), genome-wide methylation analyses revealed sex-dependent methylation clustering within W-linked regions and recombination-suppressed blocks [36]. Similarly, in Nile tilapia, sex-associated methylation differences were enriched near known sex-determining regions and genes involved in gonadal differentiation [14,37,46]. In medaka, localized epigenetic modifications around the master sex-determining gene *dmrt1bY* have been implicated in reinforcing male developmental pathways [47]. In the present study, Superscaffold20 harbored extended blocks of sex-biased methylation that flank, but do not overlap, the sex-specific coverage-differentiated interval identified in our previous genomic analysis. Although the spatial proximity of these independent signals is noteworthy, the absence of direct overlap precludes assigning this region as a primary sex determination locus based on the current evidence. Instead, these findings identify Superscaffold20 as a candidate sex-linked region that warrants further investigation. Integration of genomic, transcriptomic, and functional data will be required to determine whether these adjacent signals are related to sex determination, sex chromosome differentiation, or other sex-specific regulatory processes.

Interestingly, canonical vertebrate sex determination regulators (e.g., *sox9*, *foxl2*, *wt1*, *nr5a1*, *nr0b1*, *gata4*, *dhh*) exhibited limited differential methylation in the liver, suggesting that persistent epigenetic divergence at master regulators may not be broadly maintained in somatic tissues. Similar observations have been reported in zebrafish and Atlantic salmon, where sex-biased methylation at core pathway genes is often tissue-specific and most pronounced in gonadal or early developmental contexts rather than adult livers [40,48,49]. This pattern aligns with models of teleost sex chromosome evolution, in which early developmental regulatory shifts initiate sex fate, while later somatic tissues primarily reflect downstream physiological dimorphism rather than ongoing control of sex determination [42]. Together, our results suggest that sex-biased methylation in this species reflects both localized chromosomal differentiation, potentially marking emerging sex-linked regions and broader systemic epigenetic modulation of dimorphic physiology, with Superscaffold20 representing a candidate for the principal sex determination region.

The liver transcriptome is highly dynamic and responds to physiological, nutritional, hormonal, and environmental conditions, reflecting the central role of the liver in metabolism and growth regulation. Previous transcriptomic studies in fish, including our study of *Pelteobagrus ussuriensis*, have demonstrated sex- and growth-associated differences in hepatic gene expression and alternative splicing [50]. Within this dynamic transcriptional context, integration of gene-level methylation and transcription revealed that sex-associated epigenetic variation in the liver is concentrated within a limited subset of genes rather than broadly distributed across the genome. While most genes exhibited little or no differential methylation and remained transcriptionally stable, loci harboring extensive methylation clustering showed a markedly higher likelihood of sex-biased expression. This threshold-dependent pattern suggests that regulatory effects emerge primarily when methylation accumulates beyond a critical density, consistent with models proposing that dense DNA methylation can alter chromatin structure and transcription factor accessibility more effectively than isolated methylation events [15,51].

The enrichment of differentially expressed genes among highly methylated loci further indicates that these regions may function as epigenetic regulatory hotspots. Such architecture has been observed across vertebrates, where the majority of the methylome remains relatively stable, while dynamic methylation is restricted to genes involved in developmental and physiological regulation [52]. In the liver, an organ characterized by pronounced transcriptional plasticity, targeted epigenetic remodeling may, therefore, represent an efficient strategy for modulating gene networks without disrupting overall genomic stability.

Integration of the methylation and transcriptomic datasets revealed that genes harboring a high number of DMSs were more likely to exhibit sex-biased expression. However, the direction of methylation bias was not significantly associated with the direction of transcriptional change. This finding suggests that the relationship between DNA methylation and gene expression in the liver is more complex than a simple positive or negative correlation. The regulatory consequences of methylation may depend on genomic context, including whether methylation occurs within promoters, gene bodies, or other regulatory elements. Moreover, the observed association between DMS burden and differential expression does not establish a causal relationship. Further locus-specific and functional investigations will be necessary to determine how sex-associated methylation differences influence hepatic transcription.

A limitation of the present study is the relatively small number of biological replicates (five fish per sex). Although the samples showed consistent genome-wide methylation profiles within each sex and clear separation between females and males, this sample size may limit statistical power for detecting subtle methylation differences and may not fully capture inter-individual variation. Future studies with larger cohorts will be required to validate the sex-associated methylation patterns identified here and to further distinguish the effects of sex from variation associated with individual growth and physiological status.

## 5. Conclusions

In this study, genome-scale MethylRAD profiling of the liver in *Ancherythroculter nigrocauda* demonstrates that sex-biased DNA methylation is widespread, enriched in regulatory regions, and functionally linked to growth, metabolic, and signaling pathways underlying female-biased sexual size dimorphism. Integration with transcriptomic data supports a threshold-dependent regulatory model, in which dense methylation clustering increases the likelihood of sex-biased gene expression. Additionally, large contiguous male-biased methylation domains on Superscaffold15 and Superscaffold20 suggest emerging sex-linked regions, with Superscaffold20 representing a candidate for the primary sex determination locus. Collectively, these findings highlight DNA methylation as a key regulatory layer shaping hepatic sexual dimorphism in teleost fish.

## Figures and Tables

**Table 1:** Statistics for FspEI generated reads in all 10 samples. CCWGG represents the degenerate sequence CC(A/T)GG, where W denotes A or T according to the IUPAC nucleotide ambiguity code.

Samples	Mapping Rate (%)	Uniq Map Rate (%)	CCGG Number	CCWGG Number	Unique Loci	Filtered Reads	Raw Reads	Filter Ratio
s2(F)	91.72	44.03	8,160,945	2,551,996	1,471,823	51,712,590	53,243,673	97.12
s5(F)	90.84	44.07	8,357,885	2,456,065	1,357,214	52,499,461	53,720,008	97.73
s6(F)	90.42	43.22	4,873,373	1,579,364	1,222,380	31,129,186	32,010,326	97.25
s7(F)	89.70	42.26	5,142,483	1,714,217	1,213,348	31,400,436	32,184,017	97.57
s9(F)	91.58	45.32	8,040,412	2,432,459	1,455,823	48,451,830	49,355,861	98.17
s18(M)	93.43	49.53	6,139,575	1,154,103	1,094,040	36,468,683	37,482,840	97.29
s19(M)	93.68	47.00	11,436,134	2,701,886	1,274,981	56,314,958	57,373,824	98.15
s20(M)	94.00	49.01	7,939,402	1,206,152	1,178,372	39,390,551	40,232,687	97.91
s23(M)	92.05	47.69	5,194,237	1,661,052	1,430,230	31,924,168	32,556,203	98.06
s25(M)	92.20	48.31	6,538,954	1,959,425	1,028,439	34,864,671	35,737,834	97.55

## Data Availability

The raw data were submitted to NCBI SRA under accession number PRJNA890278.

## Supplementary Materials

The following supporting information can be downloaded at: https://www.mdpi.com/article/10.3390/ani16203300/s1, Figure S1: Genome-wide characteristics of CpG islands in the genome. (A) Fraction of symmetric motifs overlapping CpG islands (9.9%) compared with CpG-island genome coverage (2.53%), indicating enrichment in CpG-rich regions. (B) Genomic annotation of CpG islands demonstrating that 14.6% reside within promoter regions (±2 kb of TSS); Figure S2: Male and female-specific DMS on gene PTPRD. Blue dots are male-specific DMS and red dots are female-specific DMS; Table S1: Significant differentially methylated loci identified between male and female liver tissues. Each row represents a methylation locus with genomic coordinates, log_2_ fold change, raw *p*-value, and Benjamini–Hochberg adjusted *p*-value (*p*adj). Positive log_2_ fold change values indicate higher methylation levels in males, whereas negative values indicate higher methylation in females. Raw and normalized read counts for each sample are provided to facilitate assessment of methylation variability across biological replicates; Table S2: Significant 250-kb genomic bins exhibiting sex-biased DNA methylation on Superscaffold15 and Superscaffold20; Table S3: Gene-level DNA methylation profiles of canonical sex-determination regulators in liver tissue of *Ancherythroculter nigrocauda*.
